# Screening for primary aldosteronism is underutilised in patients with chronic kidney disease

**DOI:** 10.1007/s40620-022-01267-3

**Published:** 2022-02-23

**Authors:** Karanjeet Chauhan, Eitan Schachna, Renata Libianto, Jessica Ryan, Holly Hutton, Peter J. Fuller, Scott Wilson, Peter G. Kerr, Jun Yang

**Affiliations:** 1 School of Clinical Sciences, Monash University, Clayton, Australia; 2 Central Clinical School, Monash University, Clayton, Australia; 3 Department of Endocrinology, Monash Health, Clayton, Australia; 4 Endocrine Hypertension Group, Centre for Endocrinology and Metabolism, Hudson Institute of Medical Research, Level 3, Block E, Monash Medical Centre, Clayton Road, Clayton, VIC 3168 Australia; 5 Department of Nephrology, Monash Health, Clayton, Australia; 6 Department of Nephrology, Alfred Health, Melbourne, Australia

**Keywords:** Chronic kidney disease, Primary aldosteronism, Hypertension, Hypokalemia, Screening

## Abstract

**Background:**

Primary aldosteronism (PA) is the most common and potentially curable endocrine cause of secondary hypertension, and carries a worse prognosis than essential hypertension. Despite the high prevalence of hypertension in patients with chronic kidney disease (CKD), the screening rates for primary aldosteronism in CKD are unknown.

**Methods:**

In this study, we retrospectively reviewed medical records of 1627 adults who presented to the nephrology clinics of 2 tertiary hospitals in Melbourne, Australia, between 2014 and 2019. In addition to assessing the pattern of screening, we also evaluated patient-specific factors associated with the decision to test for primary aldosteronism. Patients were excluded from the final analysis if they did not have CKD, had an organ transplant, had end stage renal failure, or had insufficient data or follow-up.

**Results:**

Of the 600 patients included in the analysis, 234 (39%) had an indication for screening for primary aldosteronism based on recommendations made by the Endocrine Society. However, only 33 (14%) were tested. They were younger, had a higher mean systolic blood pressure, better renal function, and lower mean serum potassium than those who were indicated but not screened. Of the 33 screened patients, an elevated aldosterone-to-renin ratio was noted in 8 patients and a diagnosis of primary aldosteronism was made in 4 patients.

**Conclusions:**

The screening rate for primary aldosteronism is low in a CKD population, especially in patients who are older, have a lower eGFR and normal serum potassium. The consequences of undiagnosed primary aldosteronism in this select population may be substantial due to the cardiovascular and renal sequelae associated with untreated disease.

**Graphical abstract:**

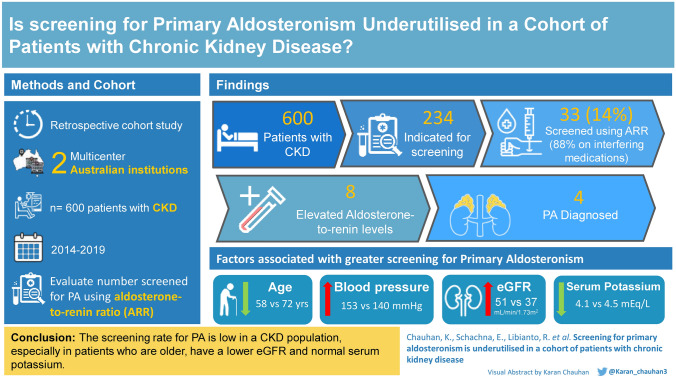

**Supplementary Information:**

The online version contains supplementary material available at 10.1007/s40620-022-01267-3.

## Introduction

Hypertension and chronic kidney disease (CKD) are interlinked major public health problems, affecting up to 34% [1] and 10% [2] of the Australian population, respectively. Worldwide, hypertension is a significant cause of CKD. In addition, up to 60–90% of individuals with chronic renal impairment (regardless of aetiology), also have hypertension [3].

The aetiology of hypertension in CKD is multifactorial. A combination of sympathetic activation, increased vascular resistance and sodium retention plays a key role in this process [3]. Poorly controlled hypertension is linked to a rapid deterioration in renal function and a disproportionately high rate of cardiovascular events, which is a leading cause of morbidity and mortality in this patient group [3]. Although the consequences of hypertension in CKD are well known, blood pressure control in this group of patients can be difficult. While the use of antihypertensives reduces morbidity and mortality, up to 40% of patients have treatment-resistant hypertension defined as inadequately controlled blood pressure (BP) despite the use of three antihypertensive agents or BP controlled on four or more medications [4].

Primary aldosteronism (PA), also called Conn syndrome, is the most common endocrine cause of secondary hypertension and is highly prevalent in patients with treatment-resistant hypertension [5]. PA is characterised by the autonomous production of aldosterone that is not suppressed by salt loading. This is most commonly due to either bilateral adrenal hyperplasia (BAH) or an aldosterone-producing adenoma (APA) [4, 5]. The autonomous production of aldosterone leads to suppressed renin and an elevated aldosterone-to-renin ratio (ARR) which is the standard screening test for PA [6, 7]. Patients with an elevated ARR subsequently undergo confirmatory testing followed by adrenal venous sampling to differentiate between unilateral disease which can be cured by adrenalectomy, and bilateral disease which can be effectively managed with targeted therapy through mineralocorticoid receptor antagonists [8, 9].

PA has long been considered a relatively benign form of hypertension. However, recent evidence has shown that patients with PA are at a 4- to 12-fold increased risk of myocardial infarction, stroke, renal impairment, and poorer quality of life when compared to patients with essential hypertension independent of blood pressure [10–13]. Aldosterone stimulates renal sodium reabsorption and volume expansion which have been found to cause direct podocyte injury through persistently increased renal perfusion and glomerular hyperfiltration [14]. Two recent studies demonstrated that the degree of renal dysfunction seen in untreated primary aldosteronism is closely linked to renal haemodynamic adaption to aldosterone excess [14, 15]. Renal biopsy specimens of 19 patients with unilateral hyperaldosteronism, compared to 22 patients with estimated glomerular filtration rate (eGFR)-matched essential hypertension, demonstrated significantly increased segmental glomerulosclerosis, interstitial fibrosis, and hyalinization of arterioles [16]. Additionally, the hyperfiltration seen in hyperaldosteronism tends to mask the severity of underlying renal damage. This is evidenced by the paradoxical drop in eGFR following PA treatment despite improvements in arterial blood pressure and the degree of proteinuria [14–17]. Targeted treatments have also been shown to reduce all-cause mortality (HR, 0.23 [CI 0.13–0.26]) [18], renal impairment [19], and cardiovascular risk [20], and to increase quality-adjusted life years [21].

**Fig. 1:**
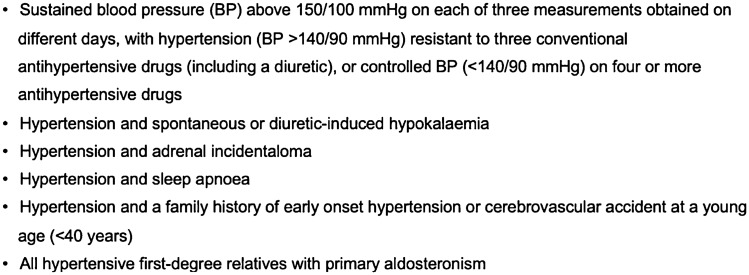
Endocrine society recommendations for PA case detection

The prevalence of PA among hypertensive patients was previously accepted at approximately 1% [22]. However, recent international literature has found it to be significantly higher at 3–13% in primary care and up to 30% in referral centres [23]. Despite the high prevalence of PA, the potential for targeted treatment and published guidelines on circumstances where PA testing is warranted (Fig. 1 [24]), the rates of screening for PA are low [25, 26]. A comparison of the recommended indications for screening and preferred testing modalities of different societies has been shown in Table 1 [27].

**Table 1:** Recommended indications for screening and testing modalities of different societies

Recommendations	Specialist guideline				
	ES	JES	SIIA	POL	SFE/SFHTA
Screening					
Hypertension	R	R	R	R	R
BP Cut-off (mmHg)	150/100	140/90	160/90	160/100	180/110
Resistant Hypertension	R	R	R	R	R
Hypertension with Adrenal incidentaloma	R	NM	R	R	R
Hypokalaemia	R	R	R	R	R
Family history of early-onset hypertension or cerebrovascular accident at a young age	R (< 40 years)	NM	R (< 50 years)	R (< 40 years)	NM
First degree relatives of patients with PA	R	NM	R	R	NM
Sleep apnoea	R	NM	NM	R	NM
Screening test of choice	Aldosterone-to-renin ratio (ARR)				
Confirmatory testing					
Saline infusion test	R	R	NM	R	R
Oral sodium loading test	R	R	NM	R	R
Urinary aldosterone (nmol/24 h)	R	R	NM	NM	NM
Captopril challenge test	R	R	NM	NM	R
Fludrocortisone suppression test	R	NM	NM	R	NM

*ES* the Endocrine Society, *JES* the Japan Endocrine Society, *SIIA* the Italian Society of Hypertension (Societa’ Italiana Dell’ Ipertensione Arteriosa), *POL* Poland, *SFE/SFHTA* the French Endocrinology Society/French Hypertension Society, *R* recommended, *NM* not mentioned

Lastly, PA testing is not specifically discussed in the current guidelines for management of hypertension in CKD and optimal strategies for diagnosing PA in CKD remain unknown even though targeted PA treatment is reno-protective [28–30]

Population-based studies of health systems in Canada and the United States have found that the screening rates for PA were less than 3% of those for whom it was recommended [25, 31]. However, similar studies have not been performed to assess screening rates for PA in patients with CKD.

We therefore set out to determine the proportion of patients with CKD attending two large tertiary nephrology outpatient clinics in Victoria, Australia, who had indications for PA screening and to assess the proportion that were appropriately screened. We also aimed to identify patient-specific factors associated with the decision by physicians to screen for PA.

## Materials and methods

### Study design and participants

In this retrospective cohort study, we assessed the medical records of 1627 consecutive patients who attended the nephrology clinics at Monash Health and Alfred Health, two major tertiary health services in Victoria, Australia, from January 2014 to April 2019.

Scanned medical records were manually assessed and data on the aetiology of CKD, diagnosis of hypertension (year, antihypertensive medication, and blood pressure measurements over 3 visits), smoking history, alcohol consumption history and comorbidities (including coronary artery disease, stroke, atrial fibrillation and left ventricular hypertrophy) were recorded. Biochemical data were extracted from the pathology databases of the respective health services.

Patients were excluded if their eGFR was greater than 60 mL/min/1.73m^2^ or if they had less than 3 blood pressure measurements during follow-up visits. We also excluded patients with end-stage renal failure defined by eGFR < 15 mL/min/1.73m^2^ and organ transplants due to the complexities associated with their management, including multiple medications which can affect aldosterone and renin status.

Approval for this project was obtained from the Human Research Ethics Committees of the respective healthcare services.

### Laboratory measurements

In general, clinical practice in the various pathology laboratories was to collect blood samples for aldosterone, renin and their ratio in the morning between 0800 and 1000 AM. Plasma aldosterone and direct renin concentrations were measured using chemiluminescent immunoassays on a DiaSorin Liaison analyser (DiaSorin, Saluggia, Italy). An ARR value ≥ 70 pmol/mU (20 ng/dL per ng/ml/h) was considered positive where plasma aldosterone concentration was measured in pmol/L and direct renin concentration in mU/L. This is equivalent to approximately 20 ng/dL per ng/mL/h when aldosterone is measured in ng/dL and renin is measured as plasma renin activity in ng/mL/h [32]. However, plasma renin activity is no longer routinely measured in most centres including our own [33].

The standard recommendation following a positive screening test was to conduct confirmatory testing using the saline suppression test. Two litres of saline was infused over 4 h and the diagnosis of PA was confirmed if the plasma aldosterone concentration remained ≥ 140 pmol/L (5.0 ng/dL) after a recumbent test or ≥ 170 pmol/L (6.1 ng/dL) after a seated test [24].

### Statistical analysis

The Kolmogorov–Smirnov test was used to assess data for normality. Continuous variables were expressed as median (interquartile range), and between-group differences were compared using the nonparametric (Mann Whitney U) test. Categorical variables were expressed as count (percentage) and comparisons between groups were analysed using the Chi-square test. A two-sided p-value of < 0.05 was considered statistically significant within a 95% confidence interval. IBM SPSS Statistics for Windows version 26 (IBM Corp., Armonk, N.Y., USA) was used to perform all statistical analyses.

## Results

### Patient demographics and clinical characteristics

**Fig. 2:**
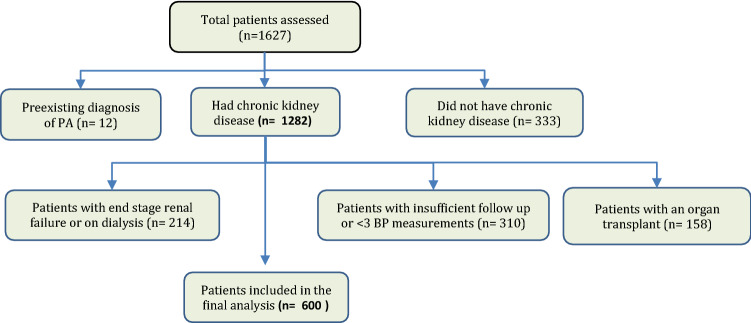
Flowchart of the selection criteria and inclusion process

Of the 1627 patients who attended the nephrology clinics, 1027 were excluded from the final analysis (Fig. 2). They included 333 patients who did not have CKD, 310 who had insufficient follow-up or less than three blood pressure measurements after their initial presentation, 158 patients who underwent an organ transplant. 12 who had a pre-existing diagnosis of PA prior to attending the nephrology clinic and 214 with end-stage renal disease or on dialysis.

Six hundred patients with CKD were included in the final analysis. Based on current PA screening recommendations made by the Endocrine Society (Fig. 2), 234 of the 600 patients (39%) met at least one criterion for PA testing while 52 (8.6%) and 5 (0.8%) met two and three criteria, respectively. Demographics and baseline data of the two groups are presented in Table 2. However, of these 234 patients in whom PA screening was indicated, only 33 (14% of 234) were screened for PA, including 21 (17% of 122) at Monash Health and 12 (11% of 112) at Alfred Health.

**Table 2:** Baseline characteristics of patients indicated for screening vs not indicated

Patient characteristics	Indicated for Screening (*n* = 234)	Not indicated for screening (*n* = 366)	P-value
Male/ Females	144/90	207/159	0.227
Median age, years	72 (57, 79)	69 (53, 78)	0.059
BMI, kg/m^2^	30 (26, 34)	28 (24, 32)	< 0.001
SBP, mmHg	140 (130, 150)	129 (122, 137)	< 0.001
DBP, mmHg	77 (73, 81)	75 (70, 80)	< 0.001
History of HTN, (%)	234 (100%)	268 (73%)	< 0.001
Duration of HTN, years	10.7 (9.4, 12.0)	10.1 (8.9, 11.4)	0.541
Alcohol consumption			0.316
Never	110 (52%)	191 (58%)	
Moderate	94 (44%)	124 (38%)	
Heavy	8 (4%)	13 (4%)	
Smoking history			0.623
Never	137 (62%)	222 (64%)	
Past smoker	59 (27%)	94 (27%)	
Current smoker	26 (12%)	32 (9%)	
eGFR, mL/min/1.73 m^2^	38 (28,50)	42 (29,55)	0.117
Creatinine, mmol/L	142 (114, 190)	134 (103, 177)	0.024
Potassium, mmol/L	4.4 (4.0, 4.9)	4.4 (4.1, 4.8)	0.757
Number of Antihypertensive	3 (2,4)	1 (1,2)	< 0.001
ACEi	77 (33%)	87 (24%)	0.014
ARB	93 (40%)	109 (30%)	0.012
Beta-blockers	125 (53%)	90 (25%)	< 0.001
MRA	36 (15%)	23 (6%)	< 0.001
Diuretics	128 (55%)	96 (26%)	< 0.001
DHP CCB	147 (63%)	93 (25%)	< 0.001
Non DHP CCB	12 (5%)	8 (2%)	0.050
History of T2DM	119 (51%)	130 (36%)	< 0.001
History of IHD	68 (29%)	73 (20%)	0.011
History of CCF	35 (15%)	30 (8%)	0.010
History of OSA	59 (25%)	7 (2%)	< 0.001
History of CVA	33 (14%)	32 (9%)	0.041
Adrenal Incidentaloma	9 (4%)	0 (0%)	< 0.001
Family history of hypertension/stroke < 40 years of age	12 (5%)	1 (0.3%)	< 0.001

*BMI* body mass index, *SBP* systolic blood pressure, *DBP* diastolic blood pressure, *eGFR* estimated glomerular filtration rate, *ACEi* angiotensin-converting enzyme inhibitor, *ARB* angiotensin receptor blocker, *MRA* mineralocorticoid receptor antagonist, *DHP CCB* dihydropyridine calcium channel blocker, *T2DM* type-2 diabetes mellitus, *IHD* ischaemic heart disease, *CCF* congestive cardiac failure, *OSA* obstructive sleep apnoea, *CVA* cerebrovascular accident; normal levels for eGFR: > 90 mL/min/1.73 m^2^; normal levels for creatinine: 60–110 mmol/L; normal levels for serum potassium: 3.5–5.0 mmol/L

The most common indications for PA screening in our cohort related to the degree of hypertension control, with resistant hypertension (n = 94), controlled hypertension on 4 or more agents (n = 70) and blood pressure greater than 150/100 (n = 46) being the top three indications.

Patients indicated for screening were significantly older with a median age of 72 years (IQR, 57–79) than patients who were not (median age 69 years; 95% CI: 53, 78) and had a significantly higher mean systolic BP of 140 mmHg (IQR, 130–150) compared to 129 mmHg (IQR, 122–137) for those not indicated for screening (Table 2). As expected, they were also on more antihypertensive agents, had more co-morbidities and a greater prevalence of hypertension (100%) than their non-indicated counterpart (73%). There was no statistically significant difference in their gender distribution, duration of hypertension, smoking history, alcohol consumption, eGFR or potassium concentration. The prevalence of hypertension in the overall cohort was 83% (268 / 300 patients from Monash Health and 230/ 300 from Alfred Health), with 46% of them being uncontrolled (BP > 140/90 mmHg). The most common comorbidities in patients with indications for PA screening were dyslipidaemia (59%), type-2 diabetes mellitus (49%), obesity (40%) and a history of acute myocardial infarction (28%).

Of the 234 patients who had indications for PA screening, 33 (14%) were screened (Table 3). They were younger, with a median age of 58 years (IQR, 45–70) compared to those who were indicated but not screened (median age 72 years [IQR, 61–81]), and had a higher mean systolic BP (153 mmHg [IQR, 138–160] vs 140 mmHg [IQR, 130, 147]) and diastolic BP (84 mmHg [IQR, 76–93] vs 77 mmHg [IQR, 73–80]).

**Table 3:** Baseline characteristics of patients with indications for screening

Patient characteristics	Indicated and screened (n = 33)	Indicated but not screened (n = 201)	P-value
Male/ Females	23/10	121/80	0.223
Median age, years	58 (45, 70)	72 (61, 81)	< 0.001
BMI, kg/m^2^	30 (26, 33)	30 (26, 34)	0.922
SBP, mmHg	153 (138, 160)	140 (130, 147)	< 0.001
DBP, mmHg	84 (76, 93)	77 (73, 80)	< 0.001
History of HTN, (%)	33 (100%)	201 (100%)	0.360
Duration of HTN, years	11.5 (6.7, 16.3)	9.7 (8.5, 11)	0.443
Alcohol consumption			0.835
Never	16 (57%)	94 (51%)	
Moderate	11 (39%)	83 (45%)	
Heavy	1 (4%)	7 (4%)	
Smoking history			0.173
Never	17 (61%)	120 (62%)	
Past smoker	5 (18%)	54 (28%)	
Current smoker	6 (21%)	20 (10%)	
eGFR, mL/min/1.73 m^2^	51 (29, 61)	37 (27, 48)	0.041
Creatinine, mmol/L	128 (94, 204)	143 (117, 191)	0.378
Potassium, mmol/L	4.1 (3.8, 4.5)	4.5 (4.1, 4.9)	0.009
Number of Antihypertensive	3 (2, 4)	3 (2, 4)	0.937
ACEi	9 (27%)	68 (34%)	0.457
ARB	11 (33%)	82 (41%)	0.417
Beta blocker	14 (42%)	111 (55%)	0.172
MRA	3 (9%)	33 (16%)	0.280
Diuretics	14 (42%)	114 (57%)	0.308
DHP CCB	24 (73%)	123 (62%)	0.216
Non DHP CCB	3 (9%)	9 (5%)	0.265
History of T2DM	10 (30%)	109 (54%)	0.011
History of IHD	5 (15%)	63 (31%)	0.058
History of CCF	3 (9%)	32 (16%)	0.308
History of OSA	7 (21%)	52 (26%)	0.568
History of CVA	7 (21%)	26 (13%)	0.205
Adrenal Incidentaloma	3 (9%)	6 (3%)	0.091
Family history of hypertension/stroke < 40 years of age	5 (15%)	7 (3%)	0.005

*BMI* body mass index, *SBP* systolic blood pressure, *DBP* diastolic blood pressure, *eGFR* estimated glomerular filtration rate, *ACEi* angiotensin-converting enzyme inhibitor, *ARB* angiotensin receptor blocker, *DHP CCB* dihydropyridine calcium channel blocker, *T2DM* type-2 diabetes mellitus, *IHD* ischaemic heart disease, *CCF* congestive cardiac failure, *OSA* obstructive sleep apnoea, *CVA* cerebrovascular accident; normal levels for eGFR: > 90 mL/min/1.73 m^2^; normal levels for creatinine: 60–110 mmol/L; normal levels for serum potassium: 3.5–5.0 mmol/L

Patients who were screened also had better renal function (mean eGFR 51 mL/min/1.73m^2^; [IQR, 29–61] vs eGFR 37 mL/min/1.73m^2^; [IQR, 27–48]), and lower mean serum potassium (4.1 mEq/L [IQR, 3.8–4.5] vs 4.5 mEq/L; [IQR, 4.1–4.9]). The most common documented causes of CKD were diabetes mellitus (31%), followed by hypertension (28%) and glomerulonephritis (15%). There was no difference in the aetiology of CKD between those who were screened and those who were not. Among the 33 patients screened for PA, 8 had an elevated ARR and 4 were diagnosed with PA. The remaining four were classified as “undefined” as they either did not undergo confirmatory testing or were lost to follow-up. Nine patients were incidentally found to have an adrenal adenoma during CT imaging as part of their renal workup. Three of these patients subsequently were tested with an ARR and a significantly high level was detected in one of them. Confirmatory testing was not performed for this particular subject and therefore a conclusive diagnosis of PA could not be established. Of the four diagnosed patients, two were biochemically and clinically cured with an adrenalectomy while two were treated with spironolactone for bilateral disease. Their eGFR decreased following targeted treatment but stabilised after 6–12 months, with baseline eGFR of 33, 54, 22 and 64 mL/min/1.73m^2^ and post-treatment eGFR of 41, 47, 16 and 39 mL/min/1.73m^2^, respectively at 12 months follow-up. The 2 patients treated medically had a suboptimal reduction in blood pressure and in the absence of renin measurements, we cannot assess whether their mineralocorticoid receptor antagonist dosage was adequate. Of the patients who were screened with an ARR, 88% (29/33) were on interfering medications. These included dihydropyridine calcium channel blockers (taken by 71%), angiotensin receptor blockers (taken by 37%), angiotensin converting enzyme inhibitors (taken by 22%) which can cause false negative results and beta blockers (taken by 38%) which can cause false positive results (Supplementary Table 1). We did not find statistically significant differences in the doses of interfering antihypertensive medications taken between those who had a positive and negative ARR (data not shown).

## Discussion

To our knowledge, this is the first study to evaluate the pattern of screening for PA as a cause of secondary hypertension in patients with CKD. Our patient cohort is representative of a typical CKD population [34] with the most common causes of CKD being diabetes mellitus, glomerulonephritis and hypertension. The prevalence of hypertension in our cohort was 83%, which is consistent with the findings of global data analysis [35].

We observed that 39% of patients had indications for PA testing at their first presentation to the nephrology clinic based on the guidelines set out by the Endocrine Society. However, less than 14% of patients who met the criteria for screening were actually tested for PA. While these rates are low, they are higher than those reported in prior studies of hypertensive groups without CKD [25, 26, 31].

Previous studies have also found that consultations with a nephrologist or endocrinologist were independently associated with a higher likelihood of screening as compared to cardiologists or general practitioners [36]. Therefore, due to the involvement of dedicated nephrologists at both study sites and the presence of an Endocrine Hypertension Service at Monash Health, it is likely that the screening rate seen in this study is an overestimate of PA screening in the wider CKD population, a large proportion of whom are managed at a primary care level [37].

We observed that a lower serum potassium concentration was independently associated with a greater likelihood of screening for PA. This is likely due to the classic presentation of PA as hypertension with hypokalemia. However, a large proportion of patients with PA have normal potassium concentrations. A study by Rossi et al. in the general population found that only 52% of patients with an aldosterone-producing adenoma and 17% of patients with bilateral adrenal hyperplasia were hypokalaemic [38], highlighting the importance of testing for PA even if potassium concentration is within normal range. One might expect this point to be even more significant for the CKD population who have a propensity for hyperkalaemia because of their underlying renal impairment. Patients with a higher eGFR and younger age were also more likely to be screened for PA. This is possibly due to the perceived benefit of PA treatment being greater in patients with less severe disease and to limited guidelines for the interpretation of aldosterone and renin levels in patients with CKD.

In keeping with other studies that highlighted delays in the diagnosis of PA [39], our study also found the median duration of hypertension to be 10.7 years in patients with indications for screening. As a result, 69% of these patients were on 3 or more antihypertensives at the time of their screening test. This complicated the interpretation of their ARR due to the effect these medications have on aldosterone and renin levels [40].

The Endocrine Society recommends that all medications that interfere with the ARR be withdrawn, in particular, mineralocorticoid receptor antagonists and other diuretics should be ceased at least 4 weeks before ARR testing [24]. A complete washout of all interfering medications or substitution with non-interfering agents is also recommended. However, due to the complexity of managing CKD, there may be a reluctance to change medications such as angiotensin receptor blockers and angiotensin converting enzyme inhibitors due to comorbid heart disease or diabetes. Additionally, re-testing after a period of washout may not always be feasible due to the time constraints encountered commonly in outpatient settings. A previous study even suggested alternative ARR thresholds for patients on interfering medications [41]. However, due to the retrospective nature of our study, we do not have enough data to comment on the necessity to withdraw interfering antihypertensive medications. This is clearly relevant as 88% of patients who underwent screening were on interfering medications. Whilst the ARR can be interpreted in the context of these confounding factors [34], the possibility of a negative screening test is rarely followed up with additional testing. The effects of different antihypertensive medications on aldosterone, renin and the aldosterone-to-renin ratio with tips on interpreting these results have been described in Table 4 [42].

**Table 4:** Effects of antihypertensives on aldosterone, renin and aldosterone-to-renin ratio

Drug	Effect on aldosterone	Effect on renin	Effect on ARR	Interpretation
ACEi	↓	↑↑	↓	Low renin is a strong predictor of PA. High renin does not exclude it
ARB	↓	↑↑	↓	Same as for ACE inhibitors
Beta-blocker	↓	↓↓	↑	Increased ARR is not clinically relevant if aldosterone is low
Calcium channel blocker (DHP)	↔ ↓	↔ ↑	↓	Discontinuation before testing is recommended
Verapamil	↔	↔	↔	Considered non-interfering
Prazosin	↔	↔	↔	Considered non-interfering
Moxonidine	↔	↔	↔	Considered non-interfering
Hydralazine	↔	↔	↔	Considered non-interfering
MRA	↔ ↑	↔ ↑↑	↔ ↓	Diagnosis of PA can be made in patients with high aldosterone and low renin. If renin is not supressed, discontinuation for 4–6 weeks is recommended before retesting
Potassium wasting diuretic	↔ ↑	↑↑	↓	Discontinuation for 4–6 weeks is recommended before testing

*ACEi* angiotensin-converting enzyme inhibitor, *ARB* angiotensin receptor blocker, *DHP* dihydropyridine, *MRA* mineralocorticoid receptor antagonist

A failure to screen for PA in indicated patients may reflect inadequate awareness of the disease or a general proclivity for treatment inertia since hypertension is both a common cause and complication of CKD [43]. The commonly used confirmatory test which requires the infusion of 2L saline over 4 h may be considered a relative contraindication in patients with severe CKD and fluid overload, and therefore serve as a deterrent to adequate PA testing. Oral salt loading poses the same risk and requires 24-h urine collection which may be inaccurate in the context of reduced urine output [44]. The best alternative test is the captopril challenge test, but it requires further validation in Caucasian populations. Ideally, patients are tested before they develop moderate to severe CKD. Another possible consideration for deficits in screening and treatment of PA in patients with CKD is the expected decline in eGFR which reflects the reversal of glomerular hyperfiltration seen in untreated hyperaldosteronism [14]. This reversal is presumed to be due to a combination of decreased BP and blockade of aldosterone-mediated hyperfiltration [45]. A study of 600 patients with treated PA demonstrated that surgical treatment was better at preventing long-term renal dysfunction than mineralocorticoid receptor antagonist therapy [19]. A recent trial by Bakris et al. found that treatment of type-2 diabetic CKD patients with finerenone, a non-steroidal, selective mineralocorticoid receptor antagonist resulted in lower risks of CKD progression and cardiovascular events [46]. However, there has also been conflicting evidence on the recommendation for use of spironolactone and other mineralocorticoid receptor antagonists in end-stage renal disease [15]. The aforementioned factors coupled with a lack of specific guidelines for diagnosing PA in patients with CKD may explain the low rate of screening. However, the exact barriers to testing for PA need to be examined in prospective studies to develop best practice guidelines in the CKD clinic.

The strengths of this study include the large, well-characterised sample of patients with CKD across two tertiary referral centres. They are representative of the typical CKD cohort based on the causes of their renal disease and prevalence of hypertension. The primary limitation of our study is the retrospective observational design resulting in the exclusion of 310 of 1627 patients due to inadequate follow-up. Of the included patients who were diagnosed with PA, few had adequate biochemical follow-up with aldosterone and renin measurements to evaluate the success of their treatment. Two patients who were treated medically for bilateral disease had a suboptimal reduction in blood pressure but the adequacy of treatment could not be assessed in the absence of follow-up renin measurements. All patients included in the analysis were referred to specialised nephrology clinics which could potentially limit the generalisability of our results to the wider CKD population, many of whom are managed within primary care [40]. For most patients, their hypertensive status and control was assessed through in-clinic BP measurements which may be prone to a greater degree of variability than standardised 24-h ambulatory blood pressure measurements. For patients who underwent screening for PA, plasma aldosterone concentration was measured using chemiluminescent immunoassays which may give falsely high values in patients with chronic renal disease due to accumulating cross-reacting steroid metabolites [47, 48]. Furthermore, patients with CKD have been found to have increasing plasma aldosterone and renin concentrations as creatinine clearance declines which could confound the interpretation of the ARR in this patient group [49, 50]. However, as demonstrated in Supplementary Table 1, patients who had ARR above the threshold of 70 pmol/mU had similar eGFR as those with ARR below 70 pmol/mU, which reinforces the clinical usefulness of the ARR as a screening tool even in the CKD population. This discrepancy could be evaluated in future studies by measuring aldosterone with liquid chromatography-tandem mass spectrometry assays (LC-​MS/MS) which have greater precision than immunoassays [47]. Finally, scanned medical records were manually assessed and all steps were taken to include the results of testing performed outside the health network if available. Any testing that was not formally documented with the treating team would have been missed, although it is unlikely that patients were evaluated for PA without the knowledge of their nephrologists.

## Conclusion

In summary, this study found that the rate of screening for primary aldosteronism, based on Endocrine Society guidelines, was low in a CKD population, especially in patients who were older, had lower eGFR and normal serum potassium. The consequences of undiagnosed primary aldosteronism in this population may be substantial due to the cardiovascular and renal sequelae associated with untreated disease. Given that 46% of our cohort had uncontrolled hypertension, it would appear necessary to screen all of these patients for primary aldosteronism with the aldosterone to renin ratio so as to facilitate the timely diagnosis of a common, treatable and potentially curable form of hypertension. A prospective study is needed to evaluate the outcomes of routine primary aldosteronism screening in patients with CKD to both understand the prevalence of primary aldosteronism and the blood pressure and renal outcomes of targeted treatment [51].

## Supplementary Information

Below is the link to the electronic supplementary material.

Supplementary file1 (DOCX 23 KB)

## Data Availability

The datasets generated and/or analysed during the current study are available from the corresponding author JY on reasonable request. The data are not publicly available due to them containing confidential patient information.
