# Supplementary material for: Screening for primary aldosteronism is underutilised in patients with chronic kidney disease
Source: J Nephrol. 2022 Feb 23;35(6):1667–77. doi: 10.1007/s40620-022-01267-3 (PMC9300536; doi:10.1007/s40620-022-01267-3)
Supplement: Supplementary file 1 — Supplementary file1 (DOCX 23 KB) [file 40620_2022_1267_MOESM1_ESM.docx]

**Supplementary table 1: Biochemical characteristics of patients who underwent testing for primary aldosteronism**

| **Patient #** | **Sex** | **Age** | **eGFR** | **K^+^** | **SBP** | **DBP** | **PAC** | **DRC** | **ARR** | **Number of interfering medications** | **Diagnosis** | **Treatment** |
| --- | --- | --- | --- | --- | --- | --- | --- | --- | --- | --- | --- | --- |
| 1 | F | 43 | 22 | 3.9 | 180 | 100 | 2290 | 17.3 | 132 | 2 | BAH | MRA |
| 2 | M | 66 | 33 | 5 | 160 | 86 | 620 | 5 | 124 | 4 | APA | Surgical resection |
| 3 | M | 62 | 54 | 4.1 | 160 | 90 | 321 | 3 | 107 | 4 | APA | Surgical resection |
| 4 | M | 78 | 17 | 3.5 | 200 | 90 | 1475 | 5 | 295 | 2 | BAH | MRA |
| 5 | F | 59 | 35 | 3.9 | 120 | 70 | 404 | 2.7 | 150 | 4 | - | - |
| 6 | M | 67 | 50 | 4.0 | 140 | 80 | 359 | 2 | 180 | 2 | - | - |
| 7 | M | 53 | 46 | 4.1 | 160 | 90 | 676 | 9.1 | 74 | 2 | - | - |
| 8 | M | 63 | 22 | 4.4 | 180 | 110 | 1660 | 11.7 | 142 | 0 | - | - |
| 9 | F | 40 | 58 | 3.6 | 200 | 100 | 180 | 16.5 | 11 | 2 | - | - |
| 10 | F | 75 | 49 | 4.9 | 145 | 70 | 2700 | 298.2 | 9 | 4 | - | - |
| 11 | F | 43 | 39 | 4.5 | 180 | 80 | 325 | 41.1 | 8 | 0 | - | - |
| 12 | M | 68 | 33 | 4.9 | 135 | 80 | 482 | 36.7 | 13 | 2 | - | - |
| 13 | M | 48 | 53 | 4.7 | 200 | 100 | 451 | 42 | 11 | 1 | - | - |
| 14 | M | 59 | 60 | 3.9 | 160 | 90 | 197 | 19.3 | 10 | 3 | - | - |
| 15 | F | 49 | 55 | 3.8 | 170 | 110 | 277 | 5.7 | 49 | 2 | - | - |
| 16 | M | 38 | 43 | 4.2 | 135 | 80 | 1110 | 20 | 56 | 3 | - | - |
| 17 | M | 73 | 17 | 4.5 | 130 | 70 | 277 | 13.2 | 21 | 4 | - | - |
| 18 | M | 44 | 43 | 4.5 | 150 | 85 | 305 | 19.5 | 16 | 1 | - | - |
| 19 | M | 68 | 34 | 4.7 | 130 | 80 | 413 | 18.3 | 23 | 0 | - | - |
| 20 | F | 46 | 45 | 4.1 | 135 | 60 | 4240 | 146.2 | 29 | 3 | - | - |
| 21 | F | 73 | 56 | 5 | 140 | 60 | 780 | 32.6 | 24 | 1 | - | - |
| 22 | M | 58 | 10 | 2.8 | 170 | 100 | 2260 | 125.7 | 18 | 2 | - | - |
| 23 | M | 53 | 28 | 3.1 | 150 | 90 | 768 | 78 | 10 | 3 | - | - |
| 24 | M | 58 | 37 | 4 | 140 | 95 | 608 | 47.8 | 13 | 3 | - | - |
| 25 | F | 39 | 44 | 4.2 | 145 | 90 | 308 | 15.5 | 20 | 2 | - | - |
| 26 | F | 80 | 49 | 3.5 | 105 | 60 | 105 | 5.9 | 18 | 4 | - | - |
| 27 | M | 57 | 34 | 4.3 | 105 | 60 | 111 | 36.4 | 3 | 2 | - | - |
| 28 | M | 79 | 33 | 4.1 | 110 | 80 | 431 | 14.8 | 29 | 2 | - | - |
| 29 | M | 59 | 54 | 3.7 | 160 | 90 | 203 | 51.6 | 4 | 2 | - | - |
| 30 | M | 62 | 54 | 5.1 | 150 | 90 | 715 | 22.2 | 32 | 3 | - | - |
| 31 | M | 36 | 51 | 4.1 | 145 | 100 | 378 | 7.2 | 53 | 2 | - | - |
| 32 | M | 78 | 23 | 4.5 | 210 | 100 | 359 | 8.3 | 43 | 1 | - | - |
| 33 | M | 54 | 57 | 3.7 | 160 | 90 | 252 | 17.7 | 14 | 0 | - | - |

eGFR, estimated glomerular filtration rate; K+, serum potassium (normal levels: 3.5-5.0 mmol/L); SBP, systolic blood pressure (mmHg); DBP, diastolic blood pressure (mmHg); PAC, plasma aldosterone concentration (pmol/L); DRC, direct renin concentration (mU/L); ARR, aldosterone-to-renin ratio (normal < 70); BAH, bilateral adrenal hyperplasia; APA, aldosterone producing adrenal adenoma; MRA, mineralocorticoid receptor antagonist
